# Supplementary material for: A mesoscopic simulator to uncover heterogeneity and evolutionary dynamics in tumors
Source: PLoS Comput Biol. 2021 Feb 10;17(2):e1008266. doi: 10.1371/journal.pcbi.1008266 (PMC7901744; doi:10.1371/journal.pcbi.1008266)
Supplement: S1 Appendix — (PDF) [file pcbi.1008266.s001.pdf]

Supporting information for:

A mesoscopic simulator to uncover heterogeneity and evolutionary dynamics in tumors

Jiménez-Sánchez, Juan[1◕], Martínez-Rubio, Álvaro[1,2,3◕], Popov, Anton[1], Pérez-Beteta, Julián[1], Azimzade, Youness[4], Molina-García, David[1], Belmonte-Beitia, Juan[1], F Calvo, Gabriel[1], Pérez-García, Víctor M[1]*,

1 Deparment of Mathematics, Mathematical Oncology Laboratory (MOLAB), Universidad de Castilla-La Mancha, Avda. Camilo José Cela, 3, 13071 Ciudad Real, Spain.
2 Department of Mathematics, Universidad de Cádiz, Avda. República Saharaui s/n, 11510 Puerto Real, Cádiz, Spain.
3 Biomedical Research and Innovation Institute of Cádiz (INiBICA), Avda. Ana de Viya 21, 11009 Cádiz, Spain
4 Department of Physics, University of Tehran, Tehran, 14395-547, Iran.

◕These authors contributed equally to this work.
* Juan.JSanchez@uclm.es

S1 Appendix. Migration process

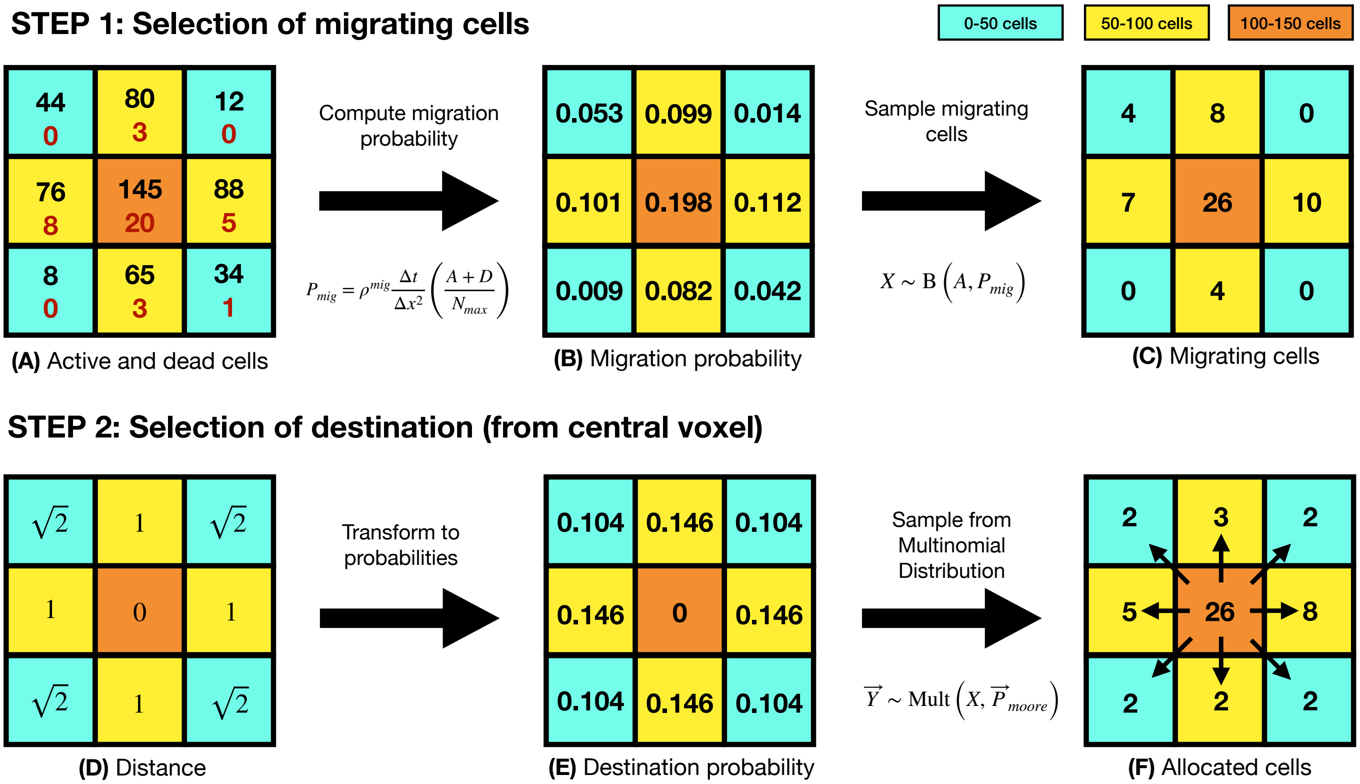

Figure 1. Example of migration process. Schematic representation of the process of migration, in a 2-dimensional domain of 9 voxels, with only one subpopulation. Notice that, in 2D, Moore neighbourhood comprises 8 voxels. **(A)** Active (black) and necrotic (red) cells in each voxel. **(B)** Probability of migration for active cells in each voxel, computed from equation X with parameters $N_{max} = 200$, $\Delta t = 24$ h, $\Delta x = 1$ mm, $\rho^{mig} = 0.01$. **(C)** Number of migrating cells, sampled from the total number of active cells according to binomial distribution. **(D)** Distance to surrounding voxels. **(E)** Probability of migration to surrounding voxels, defined as the inverse of the distance. Values are normalized to a probability measure. **(F)** Distribution of migrating cells from central voxel, according to probabilities computed in (E), sampled from multinomial distribution.
