# Supplementary material for: A mesoscopic simulator to uncover heterogeneity and evolutionary dynamics in tumors
Source: PLoS Comput Biol. 2021 Feb 10;17(2):e1008266. doi: 10.1371/journal.pcbi.1008266 (PMC7901744; doi:10.1371/journal.pcbi.1008266)
Supplement: S2 Appendix — (PDF) [file pcbi.1008266.s002.pdf]

Supporting information for:

A mesoscopic simulator to uncover heterogeneity and evolutionary dynamics in tumors

Jiménez-Sánchez, Juan[1]☯, Martínez-Rubio, Álvaro[1,2,3]☯, Popov, Anton[1], Pérez-Beteta, Julián[1], Azimzade, Youness[4], Molina-García, David[1], Belmonte-Beitia, Juan[1], F Calvo, Gabriel[1], Pérez-García, Víctor M[1]*,

1 Deparment of Mathematics, Mathematical Oncology Laboratory (MOLAB), Universidad de Castilla-La Mancha, Avda. Camilo José Cela, 3, 13071 Ciudad Real, Spain.
2 Department of Mathematics, Universidad de Cádiz, Avda. República Saharaui s/n, 11510 Puerto Real, Cádiz, Spain.
3 Biomedical Research and Innovation Institute of Cádiz (INiBICA), Avda. Ana de Viya 21, 11009 Cádiz, Spain
4 Department of Physics, University of Tehran, Tehran, 14395-547, Iran.

☯These authors contributed equally to this work.
* Juan.JSanchez@uclm.es

S2 Appendix. Bayesian optimization of mutation weights

We performed a parameter optimization in order to estimate a set of mutation weights that provide an alteration frequency resembling that of glioblastoma. There is a mutation weight for each alteration (3 considered in the model) affecting each basic process (4 processes), so we need to explore a 12-dimensional parameter space in an efficient way. In this work we resorted to Approximate Bayesian Computation (ABC) rejection algorithm, due to the lack of direct experimental measures of mutational weights. In this sense, our *ab initio* approach is not intended to provide the best 12-dimensional set of mutational weights for glioblastoma, but a reasonable guess (in the form of a probability distribution), considering that parameter exploration in 12-dimensional space may be prone to getting stuck in local minima. That is the reason why much more thorough and computationally intensive procedures were discarded for this purpose.

The ABC rejection algorithm is based on Bayes' theorem:

$$p(\theta|D) = \frac{p(D|\theta)p(\theta)}{p(D)} \tag{1}$$

where the conditional probability of a specific parameter value θ given a dataset D is related to the probability of D given the parameter value θ. Using the nomenclature of Bayesian statistics, $p(\theta|D)$ denotes the posterior, $p(D|\theta)$ the likelihood, $p(\theta)$ the prior, and $p(D)$ the evidence. The prior is understood as the probability distribution that encompasses our beliefs regarding parameter value θ before knowing D. Applied to the case of the model, our prior will be the knowledge-based guess about how alterations in glioblastoma affect basic cell processes. According to Bayes, from a reasonable prior it is possible to estimate a posterior given that we know the evidence $p(D)$ (the data that we have) and the likelihood $p(D|\theta)$ (the probability of obtaining D given the parameter value θ). The goal of ABC rejection algorithm is precisely to approximate the posterior by iteratively refining the prior.

To do so, ABC rejection algorithm first step consist on sampling N parameter points $\hat{\theta}$ from the prior, and then running one simulation per sampled parameter point with model M. This will lead to a simulated dataset $\hat{D}$, that will differ to some extent from observed dataset D. Depending on a tolerance ϵ, we accept simulations from $\hat{D}$ that keep true this criterium:

$$d(\hat{D}, D) \leq \epsilon$$

where $d(\hat{D}, D)$ is a measure of the discrepance between observed and simulated datasets. From accepted simulations we can build a posterior distribution $p(\hat{\theta}|\hat{D})$, and use it as a prior for the next iteration of the ABC algorithm. After several steps, it is expected that:

$$p(\hat{\theta}|\hat{D}) \xrightarrow{N\to\infty} p(\theta|D)$$

so that we inferred a plausible posterior solely from our prior and simulation data, using observed data as rejection criteria.

In our approach, we run a cohort of $N = 10^4$ simulations, fixing characteristic times of basic processes, and letting mutation weights be randomly chosen from a uniform distribution between 0 and 1 ($p(\theta) = \mathrm{U}(0, 1)$). The only restraint applied at this point was that the sum of mutation weights for a single process could not be greater than one (see Methods in the main text). Once all simulations had finished, we selected a diagnostic volume for all of them, randomly sampled from an empirical distribution

built with data from The Cancer Genome Atlas (TCGA) (Fig 1). The alteration frequencies at diagnostic ($\hat{D}$) were compared with their equivalents coming from TCGA data (D): 75 % of tumors sampled from TCGA have alterations in at least one gene [illegible]

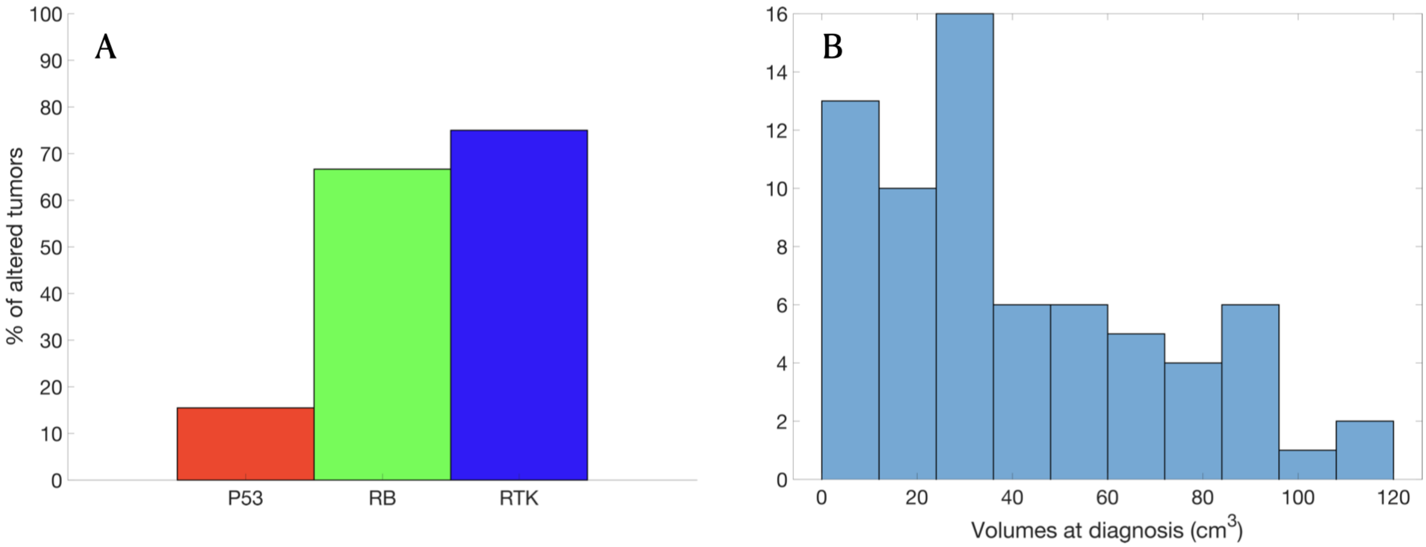

Figure 1. **Data from TCGA glioblastoma sample.** **A** Alteration frequencies of main glioblastoma pathways considered in the model. These frequencies have been measured from all TCGA glioblastoma patients. **B** Distribution of tumor volumes segmented from clinical images taken at diagnosis, from a sample of 69 tumors from TCGA open database.

The mutation weights from accepted simulations were retrieved and used to build a posterior distribution, thus providing a first coarse estimate of mutation weights for glioblastoma. The repetition of this process of random selection of weights, simulation, and rejection of non plausible tumors is intended to refine the posterior distribution of mutation weights, given that random sampling is performed over the posterior distribution obtained in previous step.

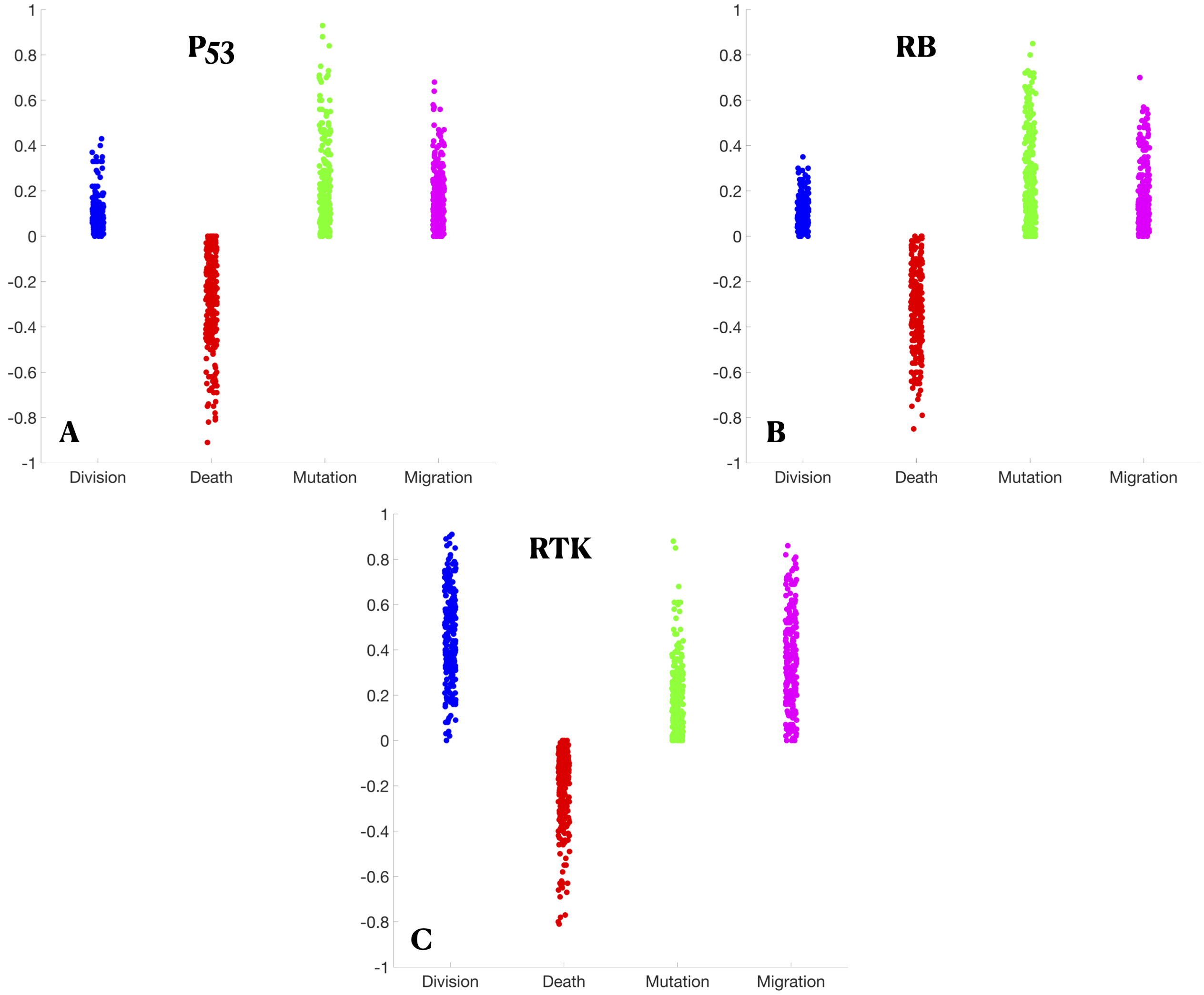

Figure 2. Mutation weights from accepted simulations. (A) Mutation weights for each process (x axis) associated to alterations in P53 pathway. Blue corresponds to cell division, red to cell death, green to mutation, and pink to migration. **(B)** Mutation weights for each process associated to alterations in Retinoblastoma pathway. **(C)** Mutation weights for each process associated to alterations in RTK/PI3K/RAS pathway. Note that weights associated to death are all negative, as alterations increase characteristic time associated to this process (instead of reducing it, as in the other processes).

In our case, we accepted 80 of 10^4 simulations in the first iteration, what makes a rejection percentage of 99.2%. With accepted simulations, we built a posterior distribution for each mutation weight. To do so, we used `ksdensity` function from Matlab. It is noteworthy that our initial guess was close to the most likely mutation weight in some cases, such as RTK weights for division and death. Moreover, attending to all posteriors, our initial guess seems plausible.

We explored the relationship between mutation weights from accepted simulations in order to uncover any hidden correlations (see Fig 4). Apart from the obvious positive self-correlation, we observed that mutation weights from different alterations affecting the same process were negatively correlated. This is a result of the implemented restraint that ensures $\sum_{i=1}^{G} w_i < 1$. Aside from that, no other strong correlations were observed.

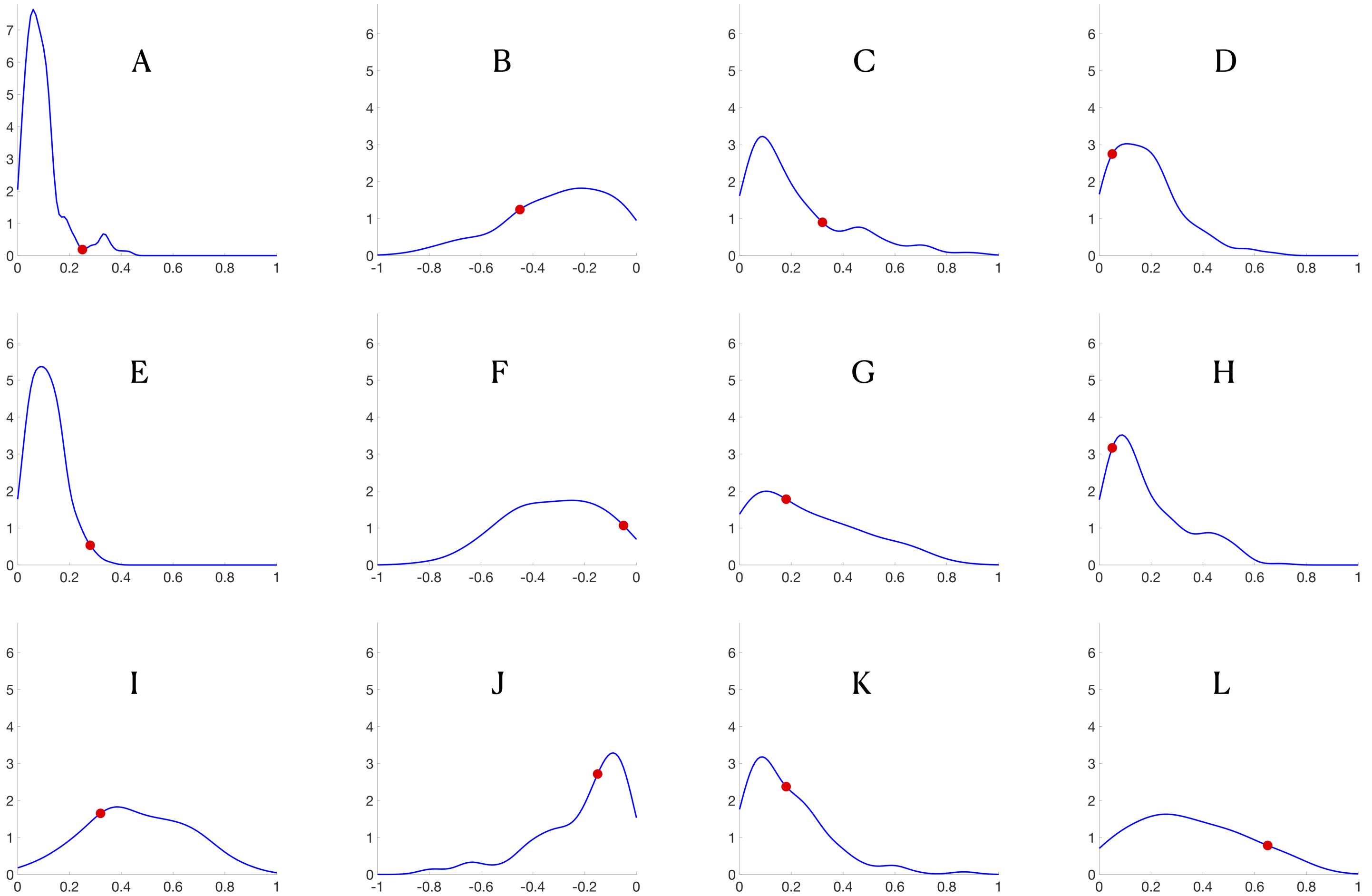

Figure 3. Posterior distributions of mutation weights from accepted simulations. Each column represents a given basic process, while each row stands for a given alteration. **(A)** Mutation weight associated to cell division of P53. **(B)** Mutation weight associated to cell death of P53. **(C)** Mutation weight associated to mutation of P53. **(D)** Mutation weight associated to migration of P53. **(E)** Mutation weight associated to cell division of RB. **(F)** Mutation weight associated to cell death of RB. **(G)** Mutation weight associated to mutation of RB. **(H)** Mutation weight associated to migration of RB. **(I)** Mutation weight associated to cell division of RTK. **(J)** Mutation weight associated to cell death of RTK. **(K)** Mutation weight associated to mutation of RTK. **(L)** Mutation weight associated to migration of RTK. Red dots denote our prior choice of weights for initial simulation tests. Note that some of our prior speculations seem consistent for the model: i.e., division and migration weights are likely small for P53 and RB, while for RTK they can take high values.

This already gives an idea about reasonable parameter values. However, in order to see the algorithm convergence, we performed a second iteration, random sampling mutation weights from previous posterior distribution. This iteration yielded 230 accepted simulations from 10^4, making a rejection percentage of 97.7%. In Fig 2 there can be seen the mutation weights of accepted simulations from second iteration, while in Fig 3 the posterior distributions obtained in second iteration are shown.

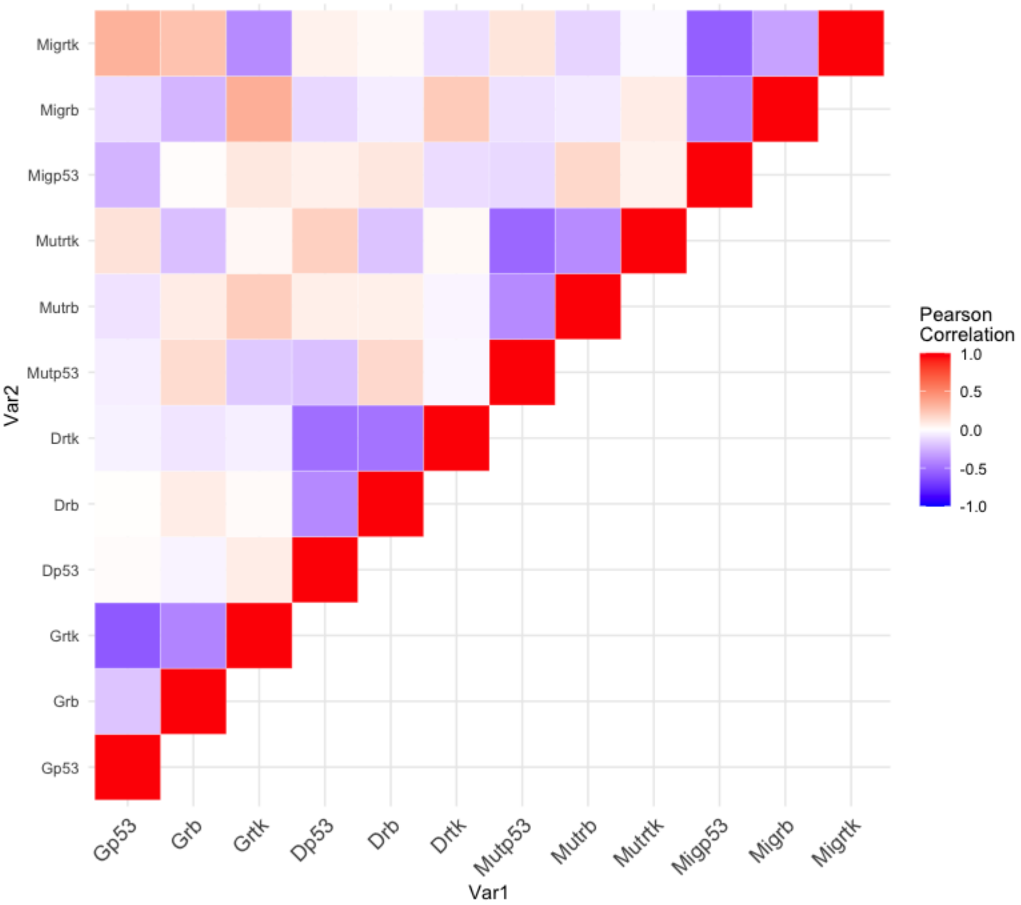

Figure 4. Correlations between mutation weights from accepted simulations. Correlation intensity is shown from strong negative (blue) to strong positive (red), with white being null correlation. Note that all correlations are slight, except for self-correlations (trivial) and correlations between weights affecting the same process (due to imposed restraints). Weights starting with 'G' affect cell division; 'D' affect cell death, 'Mut' affect mutation, and 'Mig' affect migration.
