# Supplementary material for: A mesoscopic simulator to uncover heterogeneity and evolutionary dynamics in tumors
Source: PLoS Comput Biol. 2021 Feb 10;17(2):e1008266. doi: 10.1371/journal.pcbi.1008266 (PMC7901744; doi:10.1371/journal.pcbi.1008266)
Supplement: S3 Appendix — (PDF) [file pcbi.1008266.s003.pdf]

Supporting information for:

A mesoscopic simulator to uncover heterogeneity and evolutionary dynamics in tumors

Jiménez-Sánchez, Juan[1◕], Martínez-Rubio, Álvaro[1,2,3◕], Popov, Anton[1], Pérez-Beteta, Julián[1], Azimzade, Youness[4], Molina-García, David[1], Belmonte-Beitia, Juan[1], F Calvo, Gabriel[1], Pérez-García, Víctor M[1]*,

1 Deparment of Mathematics, Mathematical Oncology Laboratory (MOLAB), Universidad de Castilla-La Mancha, Avda. Camilo José Cela, 3, 13071 Ciudad Real, Spain.
2 Department of Mathematics, Universidad de Cádiz, Avda. República Saharaui s/n, 11510 Puerto Real, Cádiz, Spain.
3 Biomedical Research and Innovation Institute of Cádiz (INiBICA), Avda. Ana de Viya 21, 11009 Cádiz, Spain
4 Department of Physics, University of Tehran, Tehran, 14395-547, Iran.

◕These authors contributed equally to this work.
* Juan.JSanchez@uclm.es

S3 Appendix. Robustness of virtual survival analysis

In this work we performed a virtual survival analysis over a cohort of 100 simulations, focusing on two macroscopic variables that possess prognostic value in real life: rim width and surface regularity. The basis for it has already been explained in the main text. However, due to the stochastic nature of some key aspects of these analysis (such as selection of diagnosis and death volumes) there are two details that need to be commented more in depth.

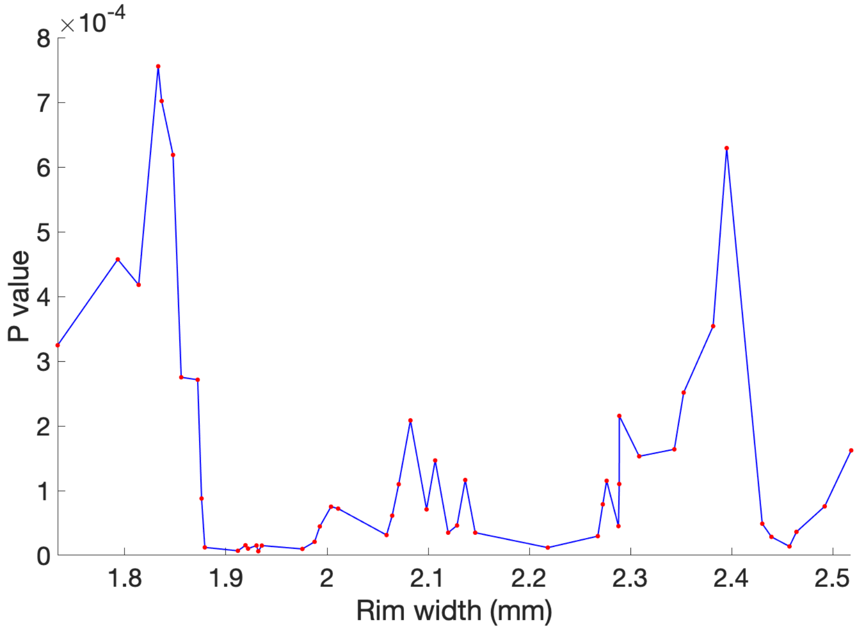

Figure 1. **P values obtained for all rim width splitting thresholds with a fixed seed.**

The first one is the selection of the best splitting threshold. Each simulation has a different value at diagnostic for macroscopic variables. From a cohort of 100 simulations we would expect to obtain 99 splitting thresholds, as many as the number of different groups we can make attending to macroscopic variables' values. However, not all splitting thresholds grant an even separation in groups. We focused on splitting thresholds producing groups where the larger was no more than 3 times the smaller. For each selected splitting threshold, we calculated corresponding Kaplan-Meier estimates, and we assessed if the separation between groups was significant using the log-rank test. In Fig 1 and Fig 2 we show splitting thresholds for rim width and surface regularity (respectively) plotted against its corresponding p-value (according to log-rank test). Notice that all splitting thresholds appear significant for rim width, while surface regularity has only two regions of significant p-values. In all cases, we select as the best splitting threshold the one with the lowest p-value, given that it is not an isolated significant splitting threshold (meaning that it is surrounded by other significant splitting thresholds). In this way we ensure that the best splitting threshold is not a random coincidence.

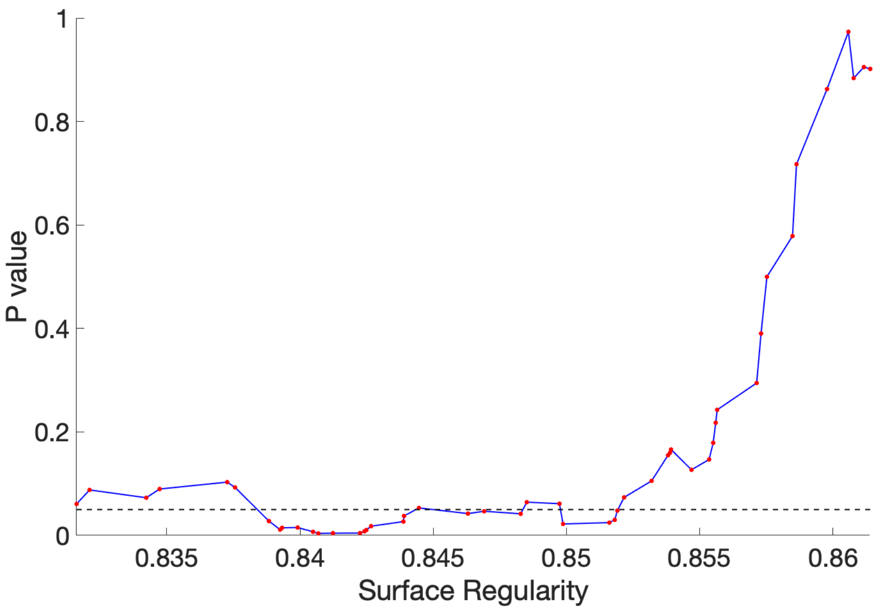

Figure 2. **P values obtained for all surface regularity splitting thresholds with a fixed seed.**

The second detail is that results do depend on random sampling of diagnostic and death volumes. If we want to assess the robustness of our results, we need to repeat them several times, varying the random seed used to randomly select diagnostic and death volumes. In doing so, the same simulation will get a different survival time for different seeds, but in this way we account for the uncertainty of diagnosis, and we overcome the model limitations regarding survival time assignment.

We repeated 1000 times the survival analysis for different seeds. Volume at diagnostic was sampled from the same empirical distribution that we used for ABC rejection algorithm (TCGA data). Additionally, we compared two different ways of sampling death volumes. For diagnostic volumes we can rely on an empirical distribution obtained from TCGA data. As little or none data is available about tumor sizes at patient death, we took as reference the work from Swanson, and we explored two different distributions for fatal tumor burden: Uniform $U(100, 120)$ and gaussian $N(110, \sqrt{10})$.

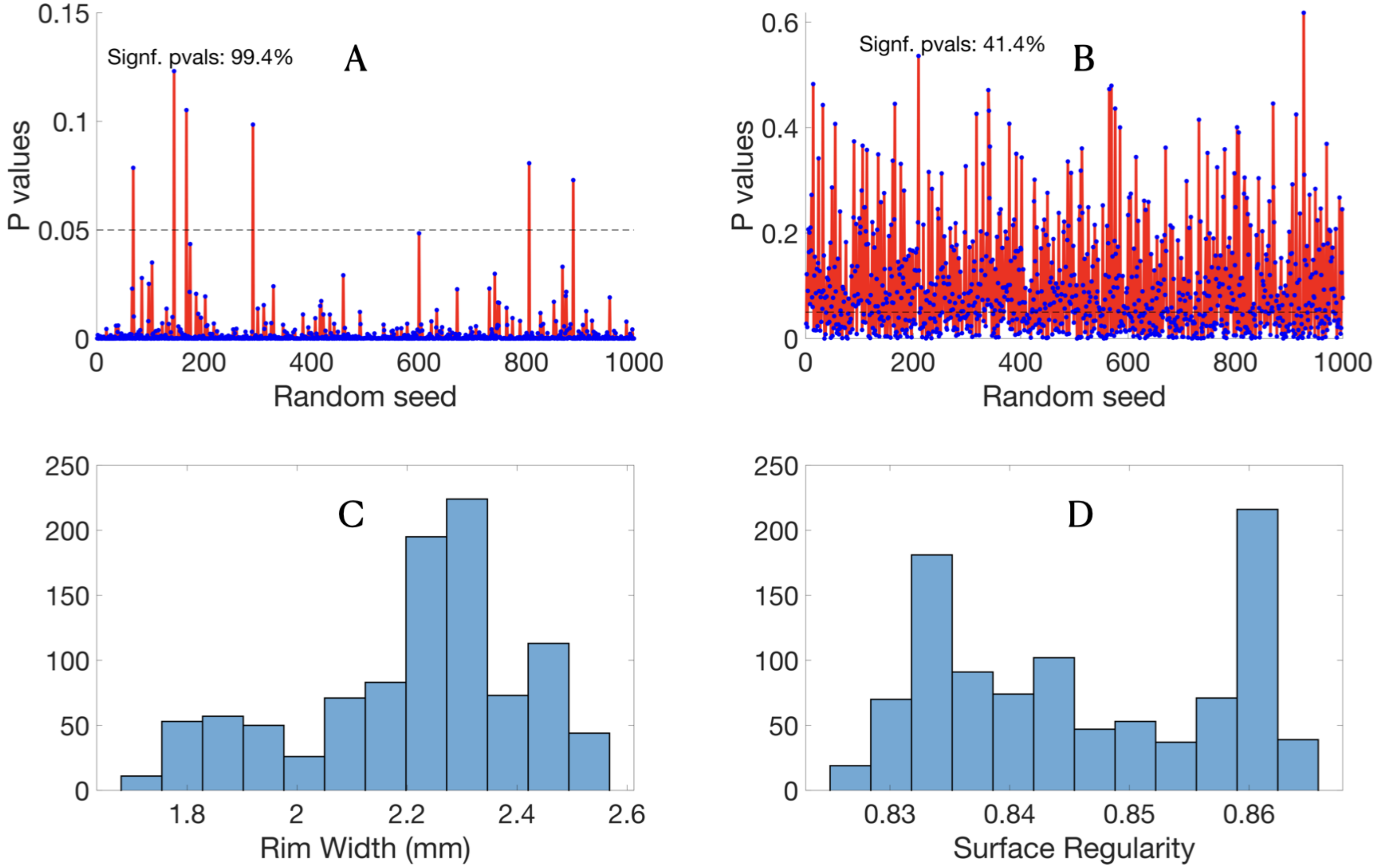

Figure 3. Robustness analysis of survival analysis with uniform sampling of death volume. A) P values from best rim width splitting thresholds for all evaluated seeds. Dotted black line represents significance threshold ($\alpha = 0.05$). **B)** P values from best surface regularity splitting thresholds for all evaluated seeds. Dotted black line represents significance threshold ($\alpha = 0.05$). **C)** Histogram of best rim width splitting thresholds for all evaluated seeds. **D)** Histogram of best surface regularity splitting thresholds for all evaluated seeds.

Robustness analysis is depicted in Fig 3 and Fig 4. In Fig 3 we can see the results coming from uniform death volume distribution, while in Fig 4 results from gaussian death volume distribution are depicted. In panels A and B from both figures we can see the p-values associated to the best splitting threshold for each evaluated seed, with A being rim width results and B being surface regularity ones. As we can see, rim width provides a significant separation between groups independently of the selected seed. This suggests that this measure is consistently calculated in the model, as its prognostic behaviour is robust across all random seeds. However, it is not the case for surface regularity, whose percentages of seeds granting significant separation between groups range from 41% (sampling death volumes from uniform distribution) to 59% (sampling death volumes from gaussian distribution). Surface regularity is a measure that greatly depends on tumor morphology, and despite our in silico tumors do have lobules and keep an irregular surface, they are mostly spherical at diagnostic volumes. Hence, the way surface regularity is calculated in the model provides almost no variability (see panel D in Fig 3 and Fig 4), with the most irregular and the most regular tumors having almost the same surface regularity. There is work to do in order to generate tumors with morphologies differing much more from a sphere. In any case, results of

surface regularity coming from gaussian death volume distribution still grant better-than-random results (59 % of significant p-values for all evaluated seeds). As best splitting thresholds' distributions for both macroscopic variables (C and D panels in Fig 3 and Fig 4) are quite similar regardless of the death volume sampling distribution, we stick to the gaussian one.

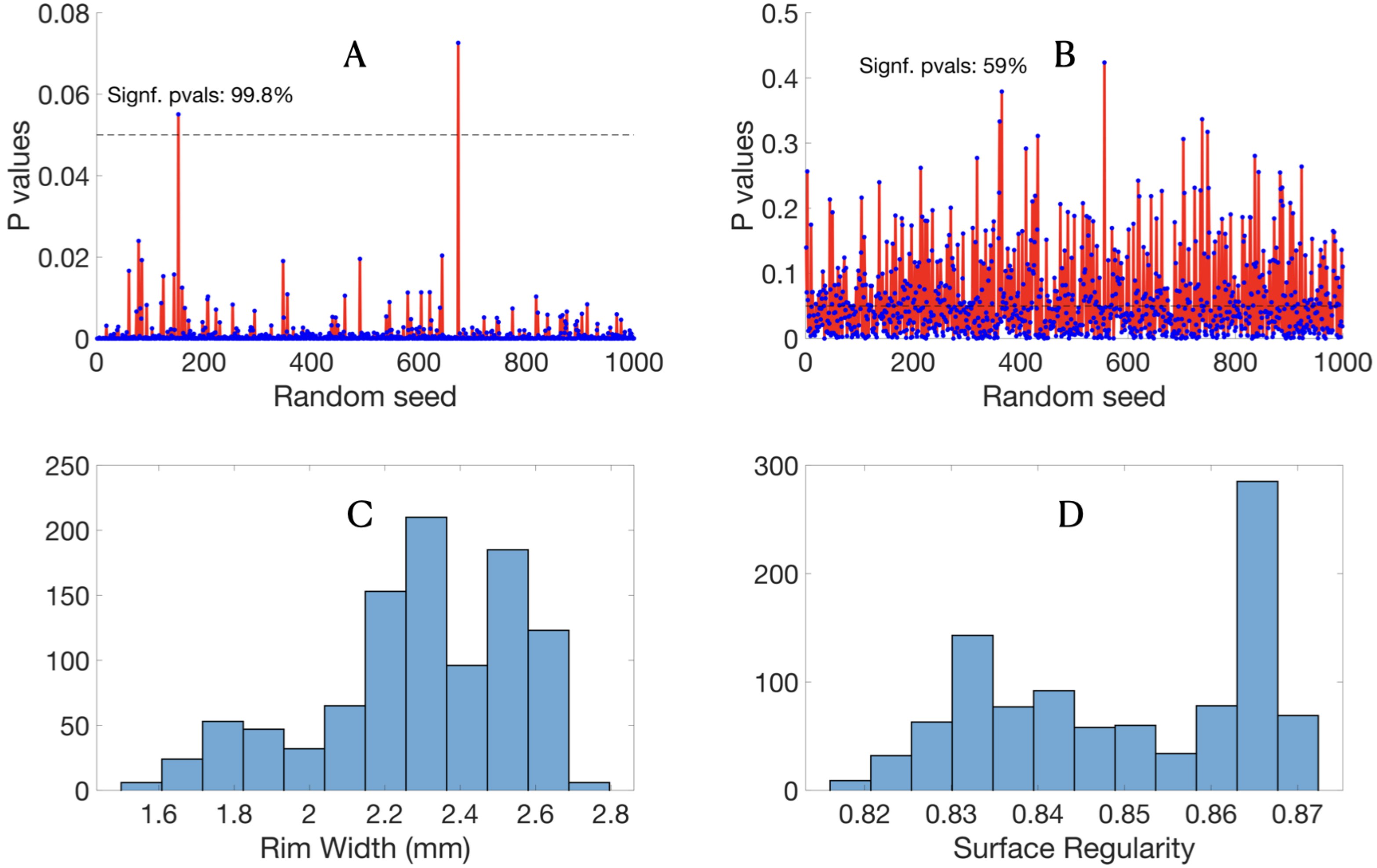

Figure 4. **Robustness analysis of survival analysis with gaussian sampling of death volume.** **A)** P values from best rim width splitting thresholds for all evaluated seeds. Dotted black line represents significance threshold ($\alpha = 0.05$). **B)** P values from best surface regularity splitting thresholds for all evaluated seeds. Dotted black line represents significance threshold ($\alpha = 0.05$). **C)** Histogram of best rim width splitting thresholds for all evaluated seeds. **D)** Histogram of best surface regularity splitting thresholds for all evaluated seeds.
