# Supplementary material for: A mesoscopic simulator to uncover heterogeneity and evolutionary dynamics in tumors
Source: PLoS Comput Biol. 2021 Feb 10;17(2):e1008266. doi: 10.1371/journal.pcbi.1008266 (PMC7901744; doi:10.1371/journal.pcbi.1008266)
Supplement: Attachment — Submitted filename: review.pdf [file pcbi.1008266.s005.pdf]

The article by Jimenez-Sanchez et al presents a stochastic mesoscopic model for tumor growth. In this model, the authors define rules in order to mimick the important cellular processes, such as proliferation ,migration, mutation and death.
They apply their modelling approach to the case of glioblastoma, and they show that the simulated tumors grow in a realistic way, first linearly and then accelerating because of the selection of more proliferating and migrating clones.
The authors studied three macroscopic quantities that have been proven to show prognostic value: surface regularity, tumor rim width and scaling exponent, and they show that in their model, as in clinics, these measures have a diagnosis value.

As the author explain, there is a lot of already existing models to describe tumor growth. However, since this model is between the macro and the microscopic ones, it has a part of originality. Moreover, the simulated tumors share several common macroscopic features with real tumors, which, at least partially, validate the model.

The main criticism is that I don't really see the need of a discrete model here. Discrete models are useful when there are a local interactions between cells or between cells and their environment for example, and when equations cannot be easily guessed. But here, there are no such local interactions. So, it seems that the evolution of the average of a lot of simulations with this model could have been reproduced by simple equations (that are very fast to be computed usually). The authors should really discuss that point.

Another important point: a model is useful if it can be used to fit clinical data. How could this model used to fit real individual tumor evolution? Would the model be able to predict the future evolution? A discussion Is also necessary here.

Other less important remarks:

- How many cells at the end? Do the simulations become slower with time, as the number of cells increases?

- Fig8: why is the threshold so precise? What happens another threshold is chosen?

- What are "are the artifacts that appear during 571 early stages of growth as a result of the discretization

- The authors also claim that their approach, the computational costs is low.: however, 5 minutes for one simulation is not so short, if you need to run 100 simulations, it takes more that 8 hours…
