# Supplementary material for: A mesoscopic simulator to uncover heterogeneity and evolutionary dynamics in tumors
Source: PLoS Comput Biol. 2021 Feb 10;17(2):e1008266. doi: 10.1371/journal.pcbi.1008266 (PMC7901744; doi:10.1371/journal.pcbi.1008266)
Supplement: Attachment — Submitted filename: ReplyLetter.pdf [file pcbi.1008266.s006.pdf]

Author Reply of Manuscript **PCOMPBIOL-D-20-01441** submitted to **PLOS Computational Biology**
Title: *A mesoscopic simulator to uncover heterogeneity and evolutionary dynamics in tumors*
Authors: J. Jiménez-Sánchez, A. Martínez-Rubio, A. Popov, J. Pérez-Beteta, Y. Azimzade, D. Molina-García, J. Belmonte-Beitia, G.F. Calvo and V.M. Pérez-García

---

Editor Comments

Thank you very much for submitting your manuscript "A mesoscopic simulator to uncover heterogeneity and evolutionary dynamics in tumors" for consideration at PLOS Computational Biology.

As with all papers reviewed by the journal, your manuscript was reviewed by members of the editorial board and by several independent reviewers. In light of the reviews (below this email), we would like to invite the resubmission of a significantly-revised version that takes into account the reviewers' comments.

We cannot make any decision about publication until we have seen the revised manuscript and your response to the reviewers' comments. Your revised manuscript is also likely to be sent to reviewers for further evaluation.

When you are ready to resubmit, please upload the following:

[1] A letter containing a detailed list of your responses to the review comments and a description of the changes you have made in the manuscript. Please note while forming your response, if your article is accepted, you may have the opportunity to make the peer review history publicly available. The record will include editor decision letters (with reviews) and your responses to reviewer comments. If eligible, we will contact you to opt in or out.

[2] Two versions of the revised manuscript: one with either highlights or tracked changes denoting where the text has been changed; the other a clean version (uploaded as the manuscript file).

Important additional instructions are given below your reviewer comments.

Please prepare and submit your revised manuscript within 60 days. If you anticipate any delay, please let us know the expected resubmission date by replying to this email. Please note that revised manuscripts received after the 60-day due date may require evaluation and peer review similar to newly submitted manuscripts.

Thank you again for your submission. We hope that our editorial process has been constructive so far, and we welcome your feedback at any time. Please don't hesitate to contact us if you have any questions or comments.

Sincerely,

Philip K. Maini
Associate Editor, PLOS Computational Biology

Douglas Lauffenburger
Deputy Editor, PLOS Computational Biology

Dear Editors,

We would like to sincerely acknowledge the three evaluators for their time, careful reading of our manuscript and for providing constructive criticism and valuable suggestions to

improve both the content and presentation of our work. We found them very helpful and below we detail, for each reviewer, our responses to their comments together with the changes made to the submitted revised version of the manuscript.

Reviewer 1

The authors present a computational framework to model cancer progression. The authors present the mathematical rationale for the various components of their simulator, and demonstrate several of the concepts and motivations of their work through an application to modeling the primary brain tumor glioblastoma. The manuscript is very well crafted, with very clear writing and visually striking figures. The authors cite much of the relevant literature, and discuss the limitations and potential future applications of their simulator. However, the authors include no data to support or validate their model. This omission leaves this reviewer confused as to whether this is a methods or results paper. The intent of the work is important in critically evaluating the suitability for publication.

Reply: We would like to thank the reviewer for his/her careful reading of the manuscript. Below we address the specific comments and questions raised.

As a methods paper, there are major concerns regarding the functionality of the codes, unit testing, comparison to other platforms, and computational benchmarking.

As a results paper, there are major concerns regarding validating and/or justifying many of the significant assumptions of the modeling platform, and reproducing the results.

Major concerns
>> The first and most important concern is to identify what is the intent of this manuscript. Is this a methods paper for a computational platform? Or a results paper?

Reply: The main point of the paper is the presentation of a computational model to study long term evolutionary dynamics in cancer. It would therefore qualify better as a methods paper, since we have explained the functionality and the algorithm. As a support for this methodology, we showed the model's capacity of recovering the tumor natural history with low computational cost and the straightforward integration with phenotypical data, while also trying to make the model readily translatable. Furthermore, we aimed at substantiating the framework by showing its applicability in clinical contexts, thereby providing analysis like those performed with actual data. In doing so, we have been able to connect geometrical characteristics and phenotypical information. This is the reason for our attempt to give hypotheses regarding geometrical features, such as the presence of more aggressive clonal populations in tumor lobules. This part of the paper might have been a source of confusion regarding the character of the work. We have clarified the intent of the paper in a new sentence in the introduction that now reads as:

'Thus, the intent of this work is to present a computational framework to study evolutionary dynamics of tumors. We provide an explanation of the functionality and access to the codes. Owing to its low computational cost and simplicity, it allows for a comparison with clinical information, revealing its potential applications in hypothesis generation and testing'.

In what follows we list our response to the reviewer's questions and comments, both referring to a 'methods' and a 'results' paper.

As a methods paper,

>> *the codes were provided as a URL to a zip file. This reviewer was unable to run the code with default installation settings in Julia. The code should be shared on a public repository such as github with more thorough documentation.*

Reply: We are sorry for the inconvenience and apologize for this. We have uploaded the codes to a public Github repository (https://github.com/JuanJS117/MesoscopicMode), adding a thorough documentation as suggested. There, we cover all required aspects to ensure that anyone can execute the code properly: from Julia installation and required packages, to parameter exploration and 3D visualization. We hope that the reviewer finds his/her concerns addressed regarding code accessibility, and we kindly ask him/her to try and download the code again and test the model.

>> *The authors use very specific probability distributions extensively throughout their approach, but provide almost no justification or rationale for their choices. Moreover, the authors do not demonstrate the sensitivity of their method/approach/results to different choices of distributions, ex. Pg 4 lines 108-109.*

Reply: The point raised by the reviewer is a key aspect of the mesoscopic model. To know how the cell number of each clonal population changes between iterations, it is necessary to account for cell gain and loss on each voxel due to the basic processes taking place in the system. Every process has two possible outcomes: success (cell division, cell death, cell migration or mutation) or failure (cell remaining still). Any process with only two possible mutually-exclusive outcomes can be regarded as a Bernoulli trial. Not all tumor cells will have the same probability of success in these processes; in fact, that probability is modulated by the characteristics of the clonal population the cell belongs to, the voxel's occupancy, and the time step between iterations (the larger the time step, the more events will take place). The discrete nature of the model assumes that cells placed in the same voxel will experience the same environmental pressures, and cells belonging to the same clonal population will have the same phenotypic characteristics. Therefore, we can assume that cells from a given clonal population in the same voxel will have the same probabilities when undergoing basic cellular processes. The discrete probability distribution that gives us the number of successes from a given number of Bernoulli trials with the same probability is the binomial distribution. Hence, by random sampling a binomial distribution with N (number of Bernoulli trials) equal to the number of cells of a given clonal population in a given voxel, and P (probability of success) equal to the shared probability calculated for a given cellular process, we get the number of cells from a specific clonal population that successfully undergo a cellular process. For each time step, this process is repeated for each clonal population, voxel and basic cellular processes considered. Thus, the binomial distribution arises naturally in this model. We have included this elaboration in the manuscript, in the 'System updating' section of Materials and Methods.

Other distributions are mentioned throughout the paper. For example, in migration events instead of two possible outcomes there are 26, so the multinomial is the natural distribution of choice. We have modified the main text and added a supplementary figure to clarify this process (Figure S1). The distributions used in the survival analysis are discussed further below in the response to another question posed by the reviewer.

>> *pg 5 line 173, What are "updating artifacts" and what is the "auxiliary structure" that mitigates them? This is an important detail to clarify, particularly for a methods paper.*

Reply: With "updating artifacts" we refer to the fact that the way in which we iterate through the grid can impact on the outcome of the simulation. This has been

thoroughly discussed in the literature (e.g. Schönfisch and de Roos, *Synchronous and asynchronous updating in cellular automata*, BioSystems, 1999;51(3):123-43). Algorithms involving fixed sweep schemes introduce additional unintended structure into the dynamics and it is preferable to choose randomly the next cell to be updated. Also, for large timesteps and populations it is reasonable to replace it by synchronous updating. With respect to the "auxiliary structure", this is an aspect of the coding of the model. We store the information of each voxel in a three-dimensional matrix. At each time step we compute and store the change in cell number of each voxel in a different matrix, since all changes are to be computed with respect to the same state (synchronous updating). After doing this for all voxels, both matrices are added and the process is restarted. This second matrix is what we refer to as auxiliary structure. Given that this is a programming aspect, and not an algorithmic property, we have removed it from the main text. We have also modified the respective paragraph and included the reference mentioned above to clarify this section.

>> *Survival analysis is dramatically oversimplified with fatal tumor burden. It is misleading to present results that imply patient survival is related to this simulation measure. This should be addressed in the Discussion and clearly stated in the Methods. This reviewer recommends–but does not insist upon–removing this analysis all together. Also a justification and reference are required for the 100cm^3.*

Reply: Patient survival in glioblastoma depends on many factors including patient age, tumor location, tumor biology and geometrical properties. However, in the context of mathematical modelling it is routine to take the volume as one of the most relevant factors as e.g. in Swanson et al. (*Virtual and real brain tumors: using mathematical modeling to quantify glioma growth and invasion*, Journal of the Neurological Sciences, 2003;216:1-10). In the context of that approximation it is customary to estimate patient survival in silico as the time it takes for the tumor to grow in diameter from the initial value to 6 cm, i.e. a volume of about 100 cm^3 (based on a sphere of volume equal to the tumor volume). We agree with the reviewer that this is a rough estimate and we have made a more realistic, yet simplified, analysis in the revised manuscript.

To do so we used diagnostic imaging data from 69 patients from the The Cancer Imaging Archive (TCIA) public database. Diagnostic GBM brain scans were segmented and their volumes computed to obtain the distribution of GBM volume sizes on diagnosis. We sampled diagnostic sizes from this distribution to perform our in silico survival analysis. To estimate the fatal tumor burden, we followed Swanson et al. estimate but allowed for a wider range of fatal tumor burden sizes, by sampling this value from a uniform distribution between 100 and 120 cm^3. We also repeated this analysis using a normal distribution centered in 110 cm^3 for fatal tumor burden. Since this analysis depends on a random sampling process, we repeated the calculations 1000 times with different seeds to assess its robustness.

The results of that study have been included in the Supporting Information and additional explanations in respective section of the main text.

>> *For code parallelization, the authors test only 2 and 4 core configurations and show in Figure 9 a non-linear relationship. The authors should test on more cores and show results. Also, how do the authors establish standard deviation for the reference 1 core simulation and why is the ST the same for all cores? These kinds of details are important for a methods paper and are effectively irrelevant for a results paper.*

Reply: This section was intended to give a glimpse of the model's parallel properties. As with many lattice gas cellular automata models, our model structure is suitable for

this kind of computational optimization. This reflects on good scalability and efficiency properties. Nonetheless, we did not perform an in-depth analysis of this computational aspect, nor its extension to GPU programming. This is in part due to the fact that the last versions of the code were affordable in terms of computational cost, with 2-6 minutes per simulation and 15-20 minutes per 100 simulations when run simultaneously from several terminals. For these reasons, we have removed this analysis from the paper altogether. Instead we have included a new figure with details on the code computational performance.

As a results paper,
>> How are the authors handling the effects of mutations on cell dynamics? The authors appear to admit that this is not measurable on pg 7 lines 202-204. If this is the case, how can the authors claim anything as a result which rests on this assumption?

Reply: We have rewritten the corresponding paragraph to explain better that the effects of mutations on cell dynamics can be qualitatively estimated from the mutations effects on the biological routes involved.

In addition to that, we have now improved substantially the estimation of the mutation weights from real data using a version of the Approximate Bayesian Computation (ABC) rejection algorithm, a well-known method suited to complex problems that arise in population genetics as described in Methods and the Supporting Information.

>> pg 7 line 214. This reviewer commends the authors for the intent to "make the study reproducible." Unfortunately however, even if the simulation codes could be run (see earlier comment), it is not clear from the very brief code documentation how the results of this study could be reproduced.

Reply: As explained before, we have now included the necessary documentation in a Github repository.

>> pg 10 lines 300, 333 the authors refer to MATLAB code, but do not provide these codes. This is important for both results and methods views of this manuscript.

Reply: Following the reviewer suggestion, we have included in the Github repository MATLAB functions that decode simulation files, retrieve basic data structure and geometric information, and generate 3D tumor renderings and MRI-like sections as those contained in the manuscript.

>> How do the authors propose to include normal tissue or stromal cells in the computational model? This is particularly important for GBM. The authors should discuss extensions to this frameworks to account for such details in future work. Also potentially aspects of the immune system and treatment.

Reply: In the revised manuscript we have now included in the discussion a paragraph explaining how to account for the description of normal tissue, immune system cells and treatments using our discrete simulation method.

>> The simulation results do not offer much insight or new findings in the "evolutionary dynamics in tumor" as the title suggests. Heterogeneity, fixation, and dominance of most aggressive clones are well known results in cancer evolutionary theory.

Reply: We hope that by having justified the methodological nature of the work will help in clarifying the point raised by the reviewer. We expect to be able to provide new emergent hypothesis coming from the framework developed here in the future.

>> pg 19 line 520 What simulation results do the authors have to claim "...competition led to natural selection..."

Reply: With this sentence we mean that a competition between different clonal populations takes place in the tumor, as all of them grow in number and expand to new voxels. The rates at which they undertake these processes vary depending on their alterations, so those with better traits will increase their fitness, thereby augmenting their abundance in the system. To help illustrate our claim, Fig. 6 B in the manuscript shows how more aggressive clonal populations (namely those that harbor more alterations) progressively increase their relative abundance in the tumor, as a result of being 'naturally selected' due to their more advantageous traits. We have changed the sentence in the discussion in order to refer more broadly to a context of evolutionary dynamics, which is the main purpose of the mesoscopic simulator.

Minor concerns and typographical errors
>> pg 3 ln 55; tumor treating fields have shown a significant improvement in GBM survival since the Stupp protocol of 2005 and should be noted.

Reply: We have changed that sentence to account for the survival improvements due to the use of TTFs.

*>> pg. 8 line 234 should be "A higher *Simpson index?"*

Reply: The reviewer is right. We have corrected this typo.

>> where does the value of 0.62 in equation 13 come from?

Reply: We have now written the exact value $\left(\frac{3}{4\pi}\right)^{1/3}$ instead of the numerical value 0.62.

>> in silico is sometimes hyphenated and sometimes not.

Reply: We thank the reviewer for spotting this. We have sticked to in "silico" (without either using the hyphenated nor the italic forms).

>> Figure 8. "Rim width" is reported to 4 significant figures of mm, which is not measurable on MRI. Report all measurements to realistic, measurable resolution.

Reply: The reviewer is right. We quoted the resulting population splitting values (both the rim width and the surface regularity) from our survival analysis, but we should have only specified the realistic figures. This has been corrected in Fig. 8.

>> Figure 9 x-axis should be in integer intervals.

Reply: This figure has now been removed from the manuscript.

*>> pg 19 line 532 "variability to *be studied."*

Reply: We thank the reviewer for detecting this typo. It has been corrected.

Reviewer 2

The article by Jimenez-Sanchez et al presents a stochastic mesoscopic model for tumor growth. In this model, the authors define rules in order to mimic the important cellular processes, such as proliferation, migration, mutation and death. They apply their modelling approach to the case of glioblastoma, and they show that the simulated tumors grow in a realistic way, first linearly and then accelerating because of the selection of more proliferating and migrating clones. The authors studied three macroscopic quantities that have been proven to show prognostic value: surface regularity, tumor rim width and scaling exponent, and they show that in their model, as in clinics, these measures have a diagnosis value.
As the authors explain, there is a lot of already existing models to describe tumor growth. However, since this model is between the macro and the microscopic ones, it has a part of originality. Moreover, the simulated tumors share several common macroscopic features with real tumors, which, at least partially, validate the model.

Reply: We would like to thank the reviewer for his/her careful reading of the manuscript and for his/her general assessment of our work.

The main criticism is that I don't really see the need of a discrete model here. Discrete models are useful when there are a local interactions between cells or between cells and their environment for example, and when equations cannot be easily guessed. But here, there are no such local interactions. So, it seems that the evolution of the average of a lot of simulations with this model could have been reproduced by simple equations (that are very fast to be computed usually). The authors should really discuss that point.

Reply: Our modelling framework is discrete, but it is also mesoscopic, on-lattice, multi-compartmental and stochastic. The key elements or agents are cell subpopulations, whose definition is parallel to how species are normally encountered in ecological models. Discretization arises by the way in which we divide the compartments, distributed on a lattice, that the cell subpopulations eventually occupy. Notice that, as stated in the manuscript, the size of those compartments is adjusted to the voxels employed in medical imaging; large enough to comprise a sufficient number of tumor cells, thus reducing strong fluctuations that would occur at the single-cell level, albeit, at the same time, small enough to provide a suitable spatial resolution of the entire tumor/tissue under consideration. This explains the feature of being mesoscopic. Furthermore, each of our voxels undergoes a dynamic (proliferation, mutation, migration and death) dictated not only by the cell subpopulations that it contains, but also by the cell subpopulations of the neighbour voxels. This dynamic is stochastic to reflect the complexity of the many interactions occurring at the cellular level.

The possibility of finding differential equations (i.e. a continuous model) resembling the model's dynamics may be far from trivial although it is a very interesting question. We do not preclude the existence of much simpler equations (e.g. a reduced set of partial differential equations involving suitable mean-field approximations) capable of reproducing what our modelling framework captures. This is in line to what was pointed out by Markham et al, *Incorporating spatial correlations into multispecies mean-field models,* Phys. Rev. E, 2013;88(5):052713. There, it was emphasized that when modelling interactions between a number of species/tumor cell populations occupying a given habitat, one often makes use of mean-field approximations, whereby spatial correlations between the locations of individuals (or subpopulations of those individuals smaller than the entire population) are neglected. Whilst this approximation holds in certain situations, this is

not always the case, and care must be taken to ensure the mean-field approximation is only used in appropriate settings. In circumstances where the mean-field approximation is unsuitable, one needs to include information on the spatial distributions of individuals (or subpopulations of those individuals), which is not a simple task. Thus, it might indeed be the case that a suitable description exist incorporating spatial information (site occupancy correlations) on the distribution of the different cell subpopulation that we consider in our framework, by means of small sets of partial differential equations, but this seems to be at present a challenging problem beyond the scope of our manuscript. Nonetheless, as suggested by the reviewer, we have included a paragraph on this point in the Discussion section.

Another important point: a model is useful if it can be used to fit clinical data. How could this model (be) used to fit real individual tumor evolution? Would the model be able to predict the future evolution? A discussion is also necessary here.

Reply: Fitting individual patients' tumors would be an ideal endpoint of this work that would require obtaining patient-specific parameter values from patient data. In general we think this is very difficult task, not only for this model but for any similar model. Rather than predicting the individual tumor evolution and response to treatments with the limited information available initially, the fitting of individual parameters, when performed on large sets of patients, may allow to perform computational studies of alternative treatment protocols. We have elaborated this in detail as suggested in the 'Discussion' section.

Also, in the revised manuscript we have used a simple version of the Approximate Bayesian Computation (ABC) rejection algorithm on a set of real GBM data, to seek for those sets of mutation weights that yield the same mutational landscapes of real tumors. This aids in having a substantially smaller sets of parameters to determine for each individual patient.

Other less important remarks:
- How many cells at the end?

Reply: Simulated tumors may reach up to 10^{10} cells. A new figure has been added to the manuscript where cell numbers at endpoint are shown for 100 simulations.

Do the simulations become slower with time, as the number of cells increases?

Reply: Simulations do become slower with time. As the tumors grow, they occupy more voxels, requiring more evaluation steps at each iteration. To make this point clear, we have included a figure in the Results section illustrating the computational aspects of the model.

Fig8: why is the threshold so precise? What happens (if) another threshold is chosen?

Reply: We have now specified realistic figures for rim width and surface regularity thresholds. A more elaborate description of the survival analysis has now been included in the Supporting Information. Briefly, we performed a search through all possible splitting thresholds, keeping those that yielded significant curve separation. The curves shown in the main text are an instance of a splitting threshold with significant median survival difference. Different thresholds offer different levels of significance and subgroup separation.

What are are the artifacts that appear during early stages of growth as a result of the discretization?

Reply: Tumors are very small during early stages of the natural history and occupy only a few voxels. Any geometrical measure estimated at those time point will be coarse and affected by noise. Thus, some quantities such as surface regularity will display strong fluctuations initially due to the discretization. For that reason, we do not take into account these variables during early stages. Clinical imaging provides tumor snapshots only after diagnosis, so that geometrical and morphological measures of real tumors can only be calculated once the tumor has reached a detectable volume above some threshold. We have explained this fact more clearly in the corresponding part of the Results section.

The authors also claim that their approach, the computational costs is low: however, 5 minutes for one simulation is not so short, if you need to run 100 simulations, it takes more that 8 hours...

Reply: The main message that we wish to convey is that the computational cost of our approach is orders of magnitude lower than those using individual-based methods, yet ours still is capable of retaining key discrete cellular properties. The possibility of parallelization or even just launching several copies of the code in multicore computers makes it easy to scale up the number of simulations run in a single computer. In our case we have run hundreds of simulations in less than one hour in multicore computers. A statement regarding computational cost of several simultaneous simulations has been added to the Results section.

Reviewer 3

In this manuscript, the authors formulate a mescoscale cellular automaton mathematical model describing the temporal and spatial evolution of tumour growth and its associated genetic composition. This model incorporates cell proliferation, death, migration, and mutations based on fixed-time probabilitistic events, allowing several events to occur in a single time step. Emphasis is given to fast computation time and ability for code parallelisation, which is a crucial component of large-scale simulations such as those performed in this manuscript. Good agreement is seen between model simulations and experimental evidence, both in terms of tumour shapes and associated growth dynamics. The model simulations predict heterogeneity in terms of tumour gene expressions, though in general, one particular gene expression tends to dominate in a local region of the tumour. Finally, a discussion concerning prognostic measures and survival analysis demonstrates a clear division between hypoactive and hyperactive tumour growth, based on typical parameter values obtained in the literature.
This paper is well written and very clearly explained. I'm impressed with the amount of novel results packed into this paper and therefore have minimal comments to suggest. One item that I do note is that some parts of the manuscript (e.g. the Discussion), while clear and informative, are long. While not essential, I would recommend some trimming of some sentences to reduce the over page count.

Reply: We would like to thank the reviewer for his/her careful reading of the manuscript and for his/her positive comments. We have trimmed non-essential sentences from the discussion as suggested. However, the total length of this section has grown due to the addition of discussion points suggested by the other reviewers.

Minor Comments:

Line 107: you have the inequality constraint that the sum of w_i is less than or equal to 1. What happens if this sum is equal to 1? Based on equation (2), and provided that the g vector is the ones vector, this means that tau_rep=0, which I don't understand how that can be used in P_rep calculations in equation (1). A similar question arises in equation (4) when sum of w^mig =1.

Reply: The reviewer is right in his/her deduction. Given that the sum of all weights w_i (for a given process) is equal to 1, the associated characteristic time τ_i would be equal to 0 (or, in the case of migration, $\rho_{\text{mig}} = 0$). This situation makes no sense in biological circumstances. Hence, weights are tuned to avoid this happening. In the simulations shown in the manuscript, weights are fixed before simulation, so we ensure that they never lead to a situation where $\tau_i = 0$. However, when searching for optimal parameters, we usually random sample weights at the start from an uniform distribution. In this case, a control must be established to avoid that $\sum_{i=1}^{G} w_i > 1$ (with G being the number of alterations). We have removed the equal sign in this equation in order to avoid confusion.

Line 109: replace 'Montecarlo' with 'Monte Carlo'

Reply: We thank the reviewer for spotting this typo. The new version of the manuscript lacks this expression after corrections due to concerns expressed by other reviewers.

Line 253: Is V=V_CE + V_I?

Reply: Yes, the sum of both magnitudes, V_{CE} and V_I represents the whole tumor volume V. This has now been made explicit in the main text.

Line 306: While Kaplan-Meier curves are mentioned in the Methods section and used in the Results (e.g. Figure 8), I don't see a mathematical definition of these survival curves listed in the Methods. Similar to how you've stated the Shannon and Simpson indices, I think that included a brief mathematical statement of what you're computing in Figure 8 would add some clarity.

Reply: We thank the reviewer for the recommendation. Following the suggestion, we included a brief explanation of the mathematical basis of Kaplan-Meier estimator, and we also mentioned the statistical test used to compare survival curves' estimates from different groups.

This concludes our reply to Manuscript **PCOMPBIOL-D-20-01441**.

Yours sincerely,

The Authors

December, 2020
